# Episodic entrainment of deep primordial mantle material into ocean island basalts

**DOI:** 10.1038/ncomms9937

**Published:** 2015-11-24

**Authors:** Curtis D. Williams, Mingming Li, Allen K. McNamara, Edward J. Garnero, Matthijs C. van Soest

**Affiliations:** 1 Department of Earth and Planetary Sciences, University of California at Davis, One Shields Avenue, Davis, California 95616, USA; 2 Arizona State University, School of Earth and Space Exploration, PO Box 876004, Tempe, Arizona 85287-6004, USA

## Abstract

Chemical differences between mid-ocean ridge basalts (MORBs) and ocean island basalts (OIBs) provide critical evidence that the Earth's mantle is compositionally heterogeneous. MORBs generally exhibit a relatively low and narrow range of ^3^He/^4^He ratios on a global scale, whereas OIBs display larger variability in both time and space. The primordial origin of ^3^He in OIBs has motivated hypotheses that high ^3^He/^4^He ratios are the product of mantle plumes sampling chemically distinct material, but do not account for lower MORB-like ^3^He/^4^He ratios in OIBs, nor their observed spatial and temporal variability. Here we perform thermochemical convection calculations which show the variable ^3^He/^4^He signature of OIBs can be reproduced by deep isolated mantle reservoirs of primordial material that are viscously entrained by thermal plumes. Entrainment is highly time-dependent, producing a wide range of ^3^He/^4^He ratios similar to that observed in OIBs worldwide and indicate MORB-like ^3^He/^4^He ratios in OIBs cannot be used to preclude deep mantle-sourced hotspots.

## Abstract (web summary)

It is unclear why some ocean island basalts at ‘hotspots' have low ^3^He/^4^He ratios similar to mid-ocean ridge basalts. Here, the authors perform convection calculations and show that these isotopic ratios can be reproduced by the episodic entrainment of deep isolated mantle reservoirs into thermal plumes.

## Graphical abstract

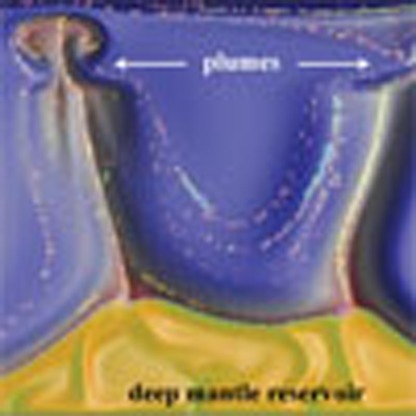

With respect to the atmospheric ratio of ^3^He/^4^He (denoted by *R*_A_), mid-ocean ridge basalts (MORBs) sampled at spreading centres not influenced by hotspot activity are globally characterized by a narrow range of values (ca. 6–11 *R*_A_), whereas ocean island basalts (OIBs) display a much wider spectrum (ca. 5–50 *R*_A_)^1^. Furthermore, the variability in OIB ^3^He/^4^He values is not only observed spatially between different hotspots, but temporally within the different-aged lavas of a single hotspot track^2^,^3^,^4^ (Fig. 1). The difference in MORB and OIB ^3^He/^4^He ratios provides evidence that they are derived from different source regions in the mantle. The relative uniformity of MORB values combined with the more variable ^3^He/^4^He ratios observed in OIB has motivated conceptual models of Earth's mantle that involve a relatively degassed, homogenous background mantle source for MORBs and a deeper, primordial (less degassed) reservoir that is tapped by mantle plumes to produce OIBs at the surface^2^,^3^. Earlier conceptual mantle models hypothesized the primordial reservoir to be a more-dense continuous layer within Earth's lowermost mantle that completely envelops the core–mantle boundary^5^,^6^. Samuel and Farnetani^6^ investigated the longevity and chemical evolution of such a basal layer and found that the mixture of materials entrained in mantle plumes can explain the wide variability of ^3^He/^4^He ratios observed in OIBs as well as the relatively uniform distribution of ^3^He/^4^He ratios observed in MORB.

In contrast to a globally continuous dense layer surrounding the entire core–mantle boundary, seismic tomography has revealed the presence of geographically discrete large low shear-wave velocity provinces in the lowermost mantle beneath Africa and the Pacific^7^,^8^,^9^ that underlie a majority of surface hotspots^10^,^11^,^12^,^13^. A principal hypothesis for the formation of the large low shear-wave velocity provinces posits that these objects represent intrinsically more dense, primordial material that has been ‘swept' into discrete thermochemical piles by convective forces of the ambient mantle^3^,^14^,^15^,^16^,^17^,^18^,^19^,^20^,^21^,^22^. These geodynamical studies reveal the presence of mantle plumes interacting with thermochemical piles, resulting in the entrainment of pile material into the plume conduits. For example, Deschamps *et al.*^21^ performed thermochemical convection calculations where plumes interacted with chemically distinct piles and, when integrated throughout Earth's history and throughout the entire upper mantle, found that the entrainment of pile material into plumes does not exceed ∼9% of the plume mass flux.

Here, we investigate whether a simple conceptual model of mantle plumes that originate in the lower mantle and interact with dense, chemically distinct primordial piles can explain the complex spatial and temporal variability of ^3^He/^4^He ratios observed in OIBs. This simplification focuses only on a primordial reservoir and neglects the possible contribution due to recycled oceanic crust^23^. In particular, this model tests the hypothesis that time-dependent entrainment of primordial material into the hottest portions of mantle plumes (that will ultimately melt near the surface) will result in changes in the ^3^He/^4^He ratio as individual hotspot tracks develop as well as differences in the ^3^He/^4^He ratio between geographically distinct hotspot tracks. Furthermore, this study investigates the dynamics of entrainment associated with both long-lived, stable mantle plumes and shorter-lived, more mobile mantle plumes, and whether these two types of plumes are expected to produce characteristically different ^3^He/^4^He signals as a function of time and space.

## Results

### Dynamic entrainment of isolated lower mantle material

This study solves the conservation equations of mass, momentum and energy in the Boussinesq approximation using an updated version of the two-dimensional Cartesian mantle convection code Citcom^24^, modified for thermochemical convection^25^. The reference case employs a Rayleigh number of 5 × 10^7^, and a viscosity that is both temperature and depth dependent. There is a 10,000 × viscosity variation between the hottest and coldest temperatures, and a 50 × viscosity step increase from upper mantle into lower mantle. The compositional field is advected using *circa* eight million tracers with the ratio tracer method^26^. The model domain represents the entire mantle thickness with a mesh that has 257 elements in the vertical direction and an aspect ratio of four. The reference model includes two compositional components: (1) background mantle that represents the source for MORBs and (2) primordial material that is ∼3% intrinsically more dense than the background mantle. More detailed model information can be found in the Methods section and McNamara *et al.*^25^.

**Figure 2:**
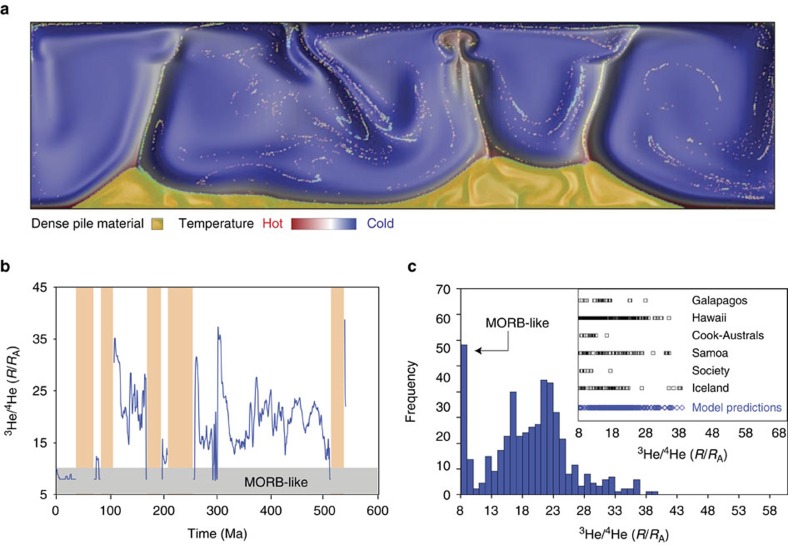
Episodic entrainment of dense material in a long-lived, stable plume. (**a**) Blue represents cold, ambient mantle material. Yellow represents compositionally distinct primordial pile material with an intrinsic density increase of 2–5% relative to the ambient mantle. (**b**) Data taken from a model with *R*_a_=5 × 10^7^, *B*=0.8, Δ*η*_660_=50, Δ*T*=3,500 K and no internal heating. ^3^He/^4^He ratios calculated using end members equivalent to MORB (^3^He/^4^He=8 *R*_A_) and OIB (^3^He/^4^He=70 *R*_A_; He concentration=100 × MORB). Tan-shaded region represents no melting. (**c**) Frequency distribution plot of model predictions from **b**. The predicted variability in ^3^He/^4^He ratios is similar to that observed in OIBs around the world (see inset).

Figure 2a shows a single snapshot in time of the calculation for the reference case, visualized as a superposition of temperature and compositional fields. Two geographically separate thermochemical piles are present in the lowermost mantle, each characterized by having thermal plumes stationed along their tops. Throughout the calculation (lasting about 600 Myr ago), the leftmost pile and plume are long lived and remain relatively unchanged (morphologically), whereas the pile and associated plumes on the right exhibit highly transient behaviour in terms of pile shape, plume flux, longevity of individual plumes and lateral fixity of plumes. Consequently, this calculation provides two characteristic types of plumes that may exist within the Earth: a long-lived plume with a steady flux of material towards the near-surface melting zone (on the left) and shorter-lived, highly mobile plumes (on the right). In all cases, the plumes entrain a small amount of the primordial pile material at discrete time steps into the centre of the plume conduit, where temperature is hottest and melting may be expected to occur.

This study first examines the source material of melt resulting from the long-lived, relatively steady plume on the left. Using the dry peridotite solidus of Hirschmann^27^, the model determines the fraction of melt that is comprised of primordial material (entrained from the piles) by comparing the compositional tracers within the numerical grid elements that have temperatures that exceed the melting temperature at a specific depth (that is, pressure). The reference case employs a 3,500 K non-adiabatic, potential temperature difference across the mantle (which is used to convert non-dimensional temperatures in the model to dimensional values). As a function of time, this model predicts that the fraction of primordial material within grid elements that exceed the melting temperature ranges from about 0 to 1% (Supplementary Fig. 1b). This model also predicts several periods of time where melting is not initiated because the dynamics of the simulation do not allow the tracers to cross the solidus. Failure to produce melts as the plume ascends results in the preservation of entrained primordial pile material that can then be transported by convective forces to other regions of melting (for example, mid-ocean ridges). Decreasing the potential temperature difference across the mantle (to 3,000 K) acts to increase both the number of time steps in which melting does not occur and the fraction of primordial material that comprises the resultant melt when melting does occur (up to 3.5% in these calculations, see Supplementary Fig. 1a) due to smaller amounts of ambient mantle material being processed during the melting event. Increasing the potential temperature difference to 4,000 K causes melting to occur more consistently throughout the length of the simulation (Supplementary Fig. 1c). In all cases, the fraction of primordial material entrained by plumes and transported into melting regions near the surface of the model is highly time-dependent but each simulation is also associated with periods of time where melting is not initiated within the plume conduit.

## Discussion

### Linking models of entrainment to real-world observations

To relate these results to actual ^3^He/^4^He ratios measured in OIBs here on Earth, the model makes the following assumptions: (1) the background (non-pile) part of the mantle is the source for average MORBs and has a globally uniform ^3^He/^4^He ratio of 8 *R*_A_. The model assumes (2) the He concentration of the primordial reservoir is 100 × higher than the background, MORB source and assigns it a ^3^He/^4^He ratio of 70 *R*_A_. While these values are not well constrained, the primordial reservoir must be characterized by a He concentration and isotopic composition greater than or equal to the highest values measured in OIBs based on mixing calculations and considerations of potential degassing^28^. Binary mixing calculations are then performed to generate Fig. 2b that displays the ^3^He/^4^He ratio of the OIB melts versus time in equivalent geologic units (Myr ago). Vertical, tan-coloured bars denote the time periods for which melting does not occur. For the reference case, the ^3^He/^4^He ratios of the melts range from 8 *R*_A_ (MORB-like) to 39 *R*_A_. Note that the pattern of this curve is identical to the curve that characterizes the fraction of primordial material within the melt (Supplementary Fig. 1b) because the assumptions of the He concentration and isotopic composition assigned to the primordial material act only to define the vertical ^3^He/^4^He axis. Therefore, it is important to highlight the character of the curve (for example, the time-dependence) over the actual isotopic values themselves.

This model predicts that as long-lived hotspot tracks develop (for example, Hawaii), their ^3^He/^4^He ratios will vary between MORB-like values and more elevated values (given the simple two-component system investigated here) as a function of both time and space (as plates migrate over relatively stationary hotspots). Drastic changes in the ^3^He/^4^He ratio within an individual hotspot can occur on very short timescales, which are similar to the rapid ^3^He/^4^He fluctuations observed at Hawaii (for example, the Hawaii Scientific Drilling Project^29^). On the other hand, constant ^3^He/^4^He values may also persist for significant periods of time depending on the characteristics of entrainment occurring in the lower mantle. Periods of time during the simulation when melting does not occur within the plume conduit results in the preservation of entrained pile material and provides a mechanism for the transport and incorporation of primordial, high ^3^He/^4^He material into the MORB source region. The results used to construct Fig. 2b are then translated into histogram form that displays ^3^He/^4^He ratio of the melt at discrete time steps throughout the calculation (Fig. 2c). For comparison, the inset within Fig. 2c shows the range of ^3^He/^4^He ratios observed in OIBs at various hotspots from the Pacific (for example, Galapagos, Hawaii, Cook-Australs, Samoa and Society). The results predicted by the simulations conducted in this study demonstrate that the highly time-dependent nature of entrainment by stable, long-lived plumes originating from the lower mantle can produce a distribution of ^3^He/^4^He ratios in melts that is consistent with that observed for OIBs here on Earth.

The numerical experiments presented here consist only of two compositional components: background MORB-source material and primordial material. Therefore, in this study the lowest possible ^3^He/^4^He ratio to characterize a melt is that which was prescribed *a priori* to the MORB-source region (8 *R*_A_). Here on Earth, many OIBs exhibit ^3^He/^4^He values lower than canonical MORB, which may be explained by the occasional addition of ancient oceanic crust within the hottest portions of mantle plumes^6^,^23^. Although not explicitly modelled here, the higher time-integrated (U+Th)/^3^He ratios of oceanic crust will lead to an enrichment of ^4^He due to radioactive decay^1^ and thereby drive the predicted ^3^He/^4^He ratios to lower values. Even with the simplification to a two-component system, these simulations predict temporally variable and complex geochemical patterns. Including additional components (for example, recycled material) would add further complexities in addition to those discussed below.

**Figure 3:**
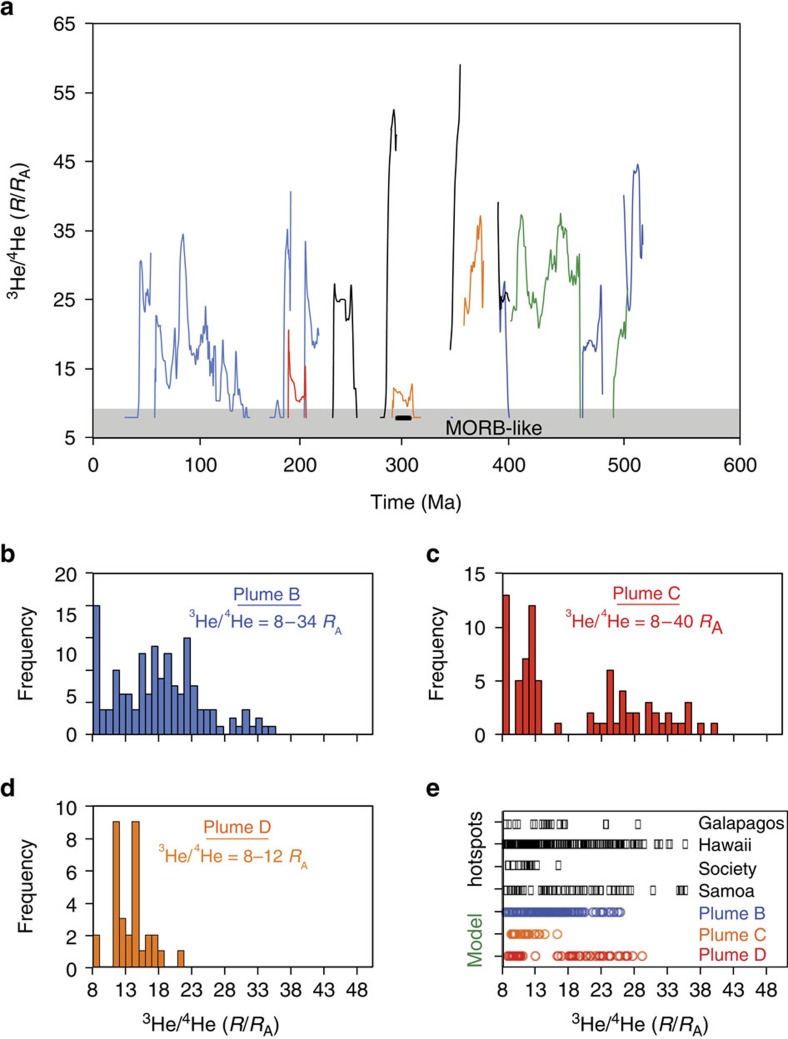
Episodic entrainment of dense material in several plumes. (**a**) Data taken from reference model (*R*_a_=5 × 10^7^, *B*=0.8, Δ*η*_660_=50, Δ*T*=3,500 K) showing time-dependent variability in the entrainment of dense, primordial pile material characterized by high ^3^He/^4^He ratios. (**b**–**d**) Frequency distribution plots that predict plumes originating from the lower mantle will be characterized by low, MORB-like ^3^He/^4^He ratios as well as elevated ^3^He/^4^He ratios. (**e**) The predicted variability in ^3^He/^4^He ratios is similar to that observed in OIBs around the world.

The plumes arising from the pile on the right side of the simulation (Fig. 2a) exhibit vastly different characteristics with respect to their duration and position due to the continual morphological changes of the right-most pile throughout the calculation. None of these plumes exist long enough to endure the entire 600 Myr ago of the calculation. This provides the opportunity to investigate plumes that may result in shorter or truncated hotspot tracks such as those observed at, for example, Samoa or the Society islands. Figure 3a shows ^3^He/^4^He ratios displayed as a function of time for each of these shorter-lived plumes (individual plumes are colour coded in Fig. 3a). Similar to the long-lived stable plume on the left side of the calculation (Fig. 2a), these plumes exhibit highly time-dependent progression of their ^3^He/^4^He ratios that range from MORB-like values of 8 *R*_A_ to values as high as 55 *R*_A_. This variability will be expressed as a function of time and space as hotspot tracks evolve and need not depend on multiple plumes sources or complex mantle dynamics. Furthermore, as shown here, MORB-like ^3^He/^4^He ratios do not necessarily indicate a shallow mantle source region and studies should proceed with caution while determining the extent of plume activity or direction of plume migration when low ^3^He/^4^He ratios are encountered. Histograms that display the distribution of ^3^He/^4^He ratios of melts sampled at each time step throughout the calculation are shown for three of these transient plumes in Fig. 3b–d. Each plume has a unique distribution of ^3^He/^4^He ratios as a function of time and space as individual hotspot tracks develop and, therefore, also in its distribution of ^3^He/^4^He ratios as shown in the histograms. Figure 3e displays the similarities in the distribution ^3^He/^4^He ratios for these three plumes compared with the measured values of OIBs at several hotspots (Galapagos, Hawaii, Society and Samoa).

This study was performed in two dimensions to facilitate higher resolution. In this geometry, plumes are actually sheets that extend infinitely in the third dimension. In three dimensions, plumes would be rooted on the cusps of piles (for example, McNamara and Zhong^17^, and Deschamps *et al.*^21^). While three-dimensional geometry may play a role in modifying the absolute magnitude of entrainment, it is not expected to change the conclusions of this study, which result from the time-dependent, discontinuous nature of entrainment of pile material into the plume. Further numerical experiments were performed to explore how the characteristics of entrainment, and by inference the ^3^He/^4^He ratio of the resultant melts, may evolve if changes in the intrinsic density (Supplementary Fig. 1) and temperature dependence of viscosity (Supplementary Fig. 2) of the primordial reservoir are made. All cases are characterized by highly time-dependent entrainment of pile material into the plume conduits, which produces near-surface melts comprised of variable fractions of primordial material. These results show that reducing or increasing the temperature dependence of viscosity tends to statistically increase or decrease the fraction of primordial material in the melt, respectively (Supplementary Fig. 2). This is easily explained by viscous coupling: the higher the temperature dependence, the less viscously coupled the two materials are, leading to reduced entrainment. This study also confirms that decreasing or increasing the density contrast between the primordial material and the background MORB source acts to statistically increase or decrease the rate of entrainment, respectively, similar to results of previous studies^30^ (Supplementary Fig. 3).

This study reveals that a simple mantle model consisting of plumes that originate in the lower mantle and physically interact with chemically distinct piles of material can produce complex, time- and space-dependent ^3^He/^4^He signatures at surface hotspots, which is compatible with observations. Furthermore, each of the plumes examined here experience episodic periods of time in which no melting event is predicted to occur because the plume is not hot enough at a specified depth (that is, pressure) in relationship to the employed solidus. In addition to providing a mechanism for the preservation and transportation of lower mantle heterogeneity through the upper mantle, this also offers a simple explanation for the formation of distinct volcanic islands within hotspot tracks. The periods of time where no melting occurs are on the order of tens of millions of years for these calculations; however, it is important to note that time is generally not well constrained in geodynamical models due to its strong sensitivity to the vigour of mantle convection that is represented by Rayleigh number. This model also predicts that, in general, a significant number of samplings will reveal no primordial material within the melt, producing melts characterized by a MORB-like ^3^He/^4^He signature. This highlights that although high ^3^He/^4^He ratios may be indicative of a deep mantle source^31^, more MORB-like ^3^He/^4^He ratios cannot be used to preclude one. Finally, the simulations presented here demonstrate that a single primordial reservoir can yield multiple mantle plumes, each having a unique ^3^He/^4^He signature that may evolve as a function of both time and space.

## Methods

### Governing equations for mantle convection calculations

The geodynamic calculations are performed by solving the non-dimensional equations for conservation of mass, momentum and energy using the Boussinesq approximation where:


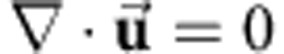



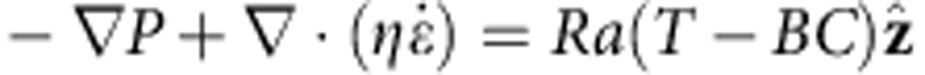



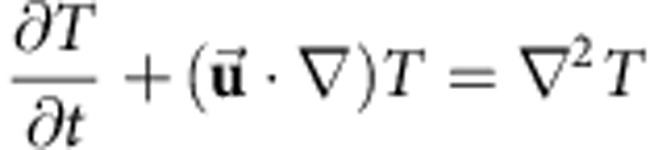


Where 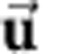 is velocity, *P* is dynamic pressure, *η* is viscosity, 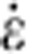 is the strain rate, *T* is the temperature, *C* is composition, 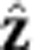 is positive in the upward vertical direction and *t* is time. The thermal Rayleigh number is defined as:


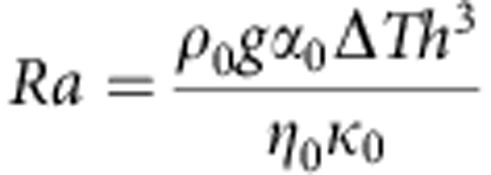


where *ρ*_0_, *α*_0_, Δ*T*, *η*_0_, and *κ*_0_ are dimensional reference values of density, thermal expansivity, the temperature difference between the core and the surface, upper mantle viscosity at a non-dimensional temperature of *T*=0.5, and thermal diffusivity, respectively. The constants *g* and *h* represent gravitational acceleration and mantle thickness, respectively. A Rayleigh number of 5 × 10^7^ is employed, referenced to upper mantle viscosity at temperature *T*=0.5. This value of Rayleigh number is consistent with values typically used for mantle convection problems (*ρ*_0_∼3,500 kg m^−3^, *g*∼10 m s^−2^, *α*_0_∼10^−5^ K^−1^, Δ*T*∼3,500 K, *η*_0_∼5 × 10^20^ Pa s, *κ*_0_∼10^−6^ m^2^ s).

The buoyancy number *B* is defined as the ratio between a chemical density anomaly and a density anomaly due to thermal expansion:


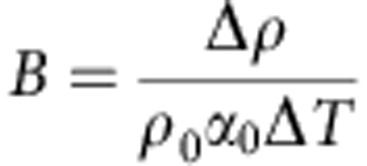


where Δ*ρ* is the density contrast between particular compositional components. Both *α*_0_ and *ρ*_0_ are constant throughout the model. In this study, we use *α*_0_=1*e*−5 and Δ*T*=3,500 K for non-dimensionalization. For a given buoyancy number, one cannot specify Δ*ρ*/*ρ*_0_ with a higher certainty than *α*_0_ Δ*T*. Here we assume that *α*_0_ Δ*T* ranges from 0.025 to 0.05, allowing for uncertainties in *α* and Δ*T*. This range of uncertainty was determined by assuming that *α* is ∼1.0 × 10^−5^ to 1.5 × 10^−5^ K^−1^ and the potential temperature, Δ*T*, is ∼3,000–4,000 K. Each calculation employed prescribed values of *B*; therefore, we state density contrast as a range of values in order to reflect this range in *α*Δ*T*.

The viscosity *η* is a function of temperature, depth and composition, and includes a viscosity increase at the base of the transition zone (equivalently at 660 km depth):


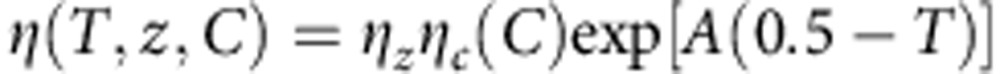


where *η*_*z*_ and *z* are non-dimensional viscosity and dimensional depth, respectively. *η*_*C*_(*C*) is the viscosity coefficient, particular to a given compositional species, while *A* and *T* are the activation parameter and non-dimensional temperature, respectively. An *A*=9.2103 leads to a viscosity range of 10^4^ due to the temperature difference between the hottest and coldest regions of the model.

Temperature boundary conditions are isothermal on the top and bottom and insulating on the sides. Velocity boundary conditions are free-slip on all boundaries. The model domain has an aspect ratio of four and the numerical grid incorporates ∼260,000 elements; 1,025 in the horizontal direction and 257 in the vertical direction. We used *circa* eight million tracers to track the compositional field using the ratio tracer method (for example, Tackley and King^26^). The grid resolution and tracer density is identical to that used in McNamara *et al.*^25^ to study small-scale ultra low velocity zones in a whole-mantle context. Resolution tests in that study confirmed that the resolution and number of tracers is appropriate to resolve small-scale features such as those that we study here. The initial condition was established by performing a series of calculations on a two-component thermochemical system consisting of only background mantle and compositional reservoir material. To aid the system towards reaching a quasi-steady thermal state, simulations were started by using a high buoyancy number for the compositional reservoir material, resulting in a purely layered system. This calculation was performed for many lifetimes of the Earth until it reached equilibrium. Using that layered convection thermal field, we introduced eight million tracers to represent the compositional reservoirs with a buoyancy number of 0.8 and then performed a series of successive thermochemical calculations, each at higher resolution. At each step of increased resolution, models were allowed to reach a quasi-steady thermal state until reaching the resolution that was ultimately used for these calculations.

Each tracer within the simulation carries with it specific information including model time, composition, temperature and depth. Melting was simulated by converting depth to pressure using PREM and then employing the dry peridotite solidus of Hirschmann^27^. Although chemically and mineralogically distinct components will most likely require their own distinct solidi, these solidi are currently not well constrained. Therefore, we have chosen to employ a single solidus to all materials in the calculations. The potential temperature difference between the hottest and coldest regions of the reference model is 3,500 K. Varying the potential temperature difference does not significantly influence the dynamics, including the amount of primordial entrainment into plumes, because it only weakly controls the Rayleigh number. However, it will modify the volume of plume material that undergoes melting. We find that increasing the temperature difference provides a larger thermal ‘halo' around the plume material resulting in more ambient material being incorporated into the melt (Supplementary Fig. 1). We have not explored lower values, but expect that they will lead to primordial material contributing a higher percentage to the overall plume melt, thereby making the time-dependent variability we are investigating even more pronounced.

For comparison with the model predictions, ^3^He/^4^He ratios of MORBs and OIBs were compiled from the GEOROC database (http://georoc.mpch-mainz.gwdg.de/), the PetDB database (http://www.petdb.org) and the USGS noble gas database (http://pubs.usgs.gov/ds/2006/202/). Only analyses conducted by *in vacuo* crushing techniques were considered to limit potential affects from cosmic ray spallation. This ‘filtering' of the database results in the removal of samples with unusually high ^3^He/^4^He ratios. In addition, low He concentrations are susceptible to contamination by air as well as having their ^3^He/^4^He ratios diminish by radiogenic ingrowth of ^4^He after eruption due to the decay of U and Th. In this context, these values should be thought of as minimum values.

## Additional information

**How to cite this article:** Williams, C. D. *et al.* Episodic entrainment of deep primordial mantle material into ocean island basalts. *Nat. Commun.* 6:8937 doi: 10.1038/ncomms9937 (2015).

## Supplementary Material

Supplementary Information Supplementary Figures 1-3

## Figures and Tables

**Figure 1:**
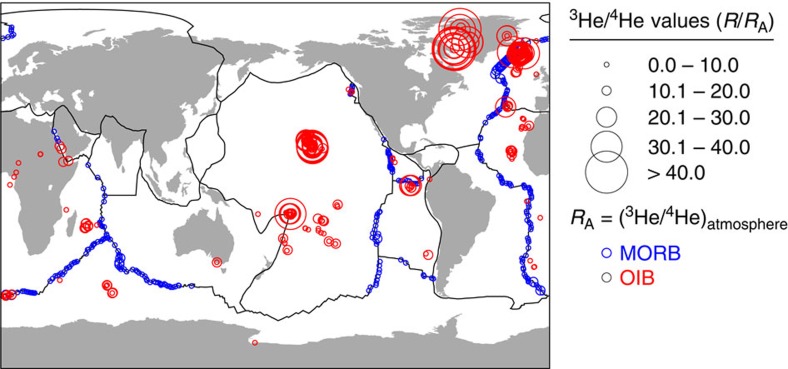
Global distribution of ^3^He/^4^He ratios in volcanic systems. Blue circles represent MORB sample locations with ^3^He/^4^He ratios ca. 8 *R*_A_. Red circles are centred at OIB sample locations and display ^3^He/^4^He ratios from MORB-like values to more elevated values. Compilation from the GEOROC (http://georoc.mpch-mainz.gwdg.de/), PetDB (http://www.petdb.org) and USGS noble gas (http://pubs.usgs.gov/ds/2006/202/) databases.
