# Supplementary material for: Episodic entrainment of deep primordial mantle material into ocean island basalts
Source: Nat Commun. 2015 Nov 24;6:8937. doi: 10.1038/ncomms9937 (PMC4673794; doi:10.1038/ncomms9937)
Supplement: Supplementary Information — Supplementary Figures 1-3 [file ncomms9937-s1.pdf]

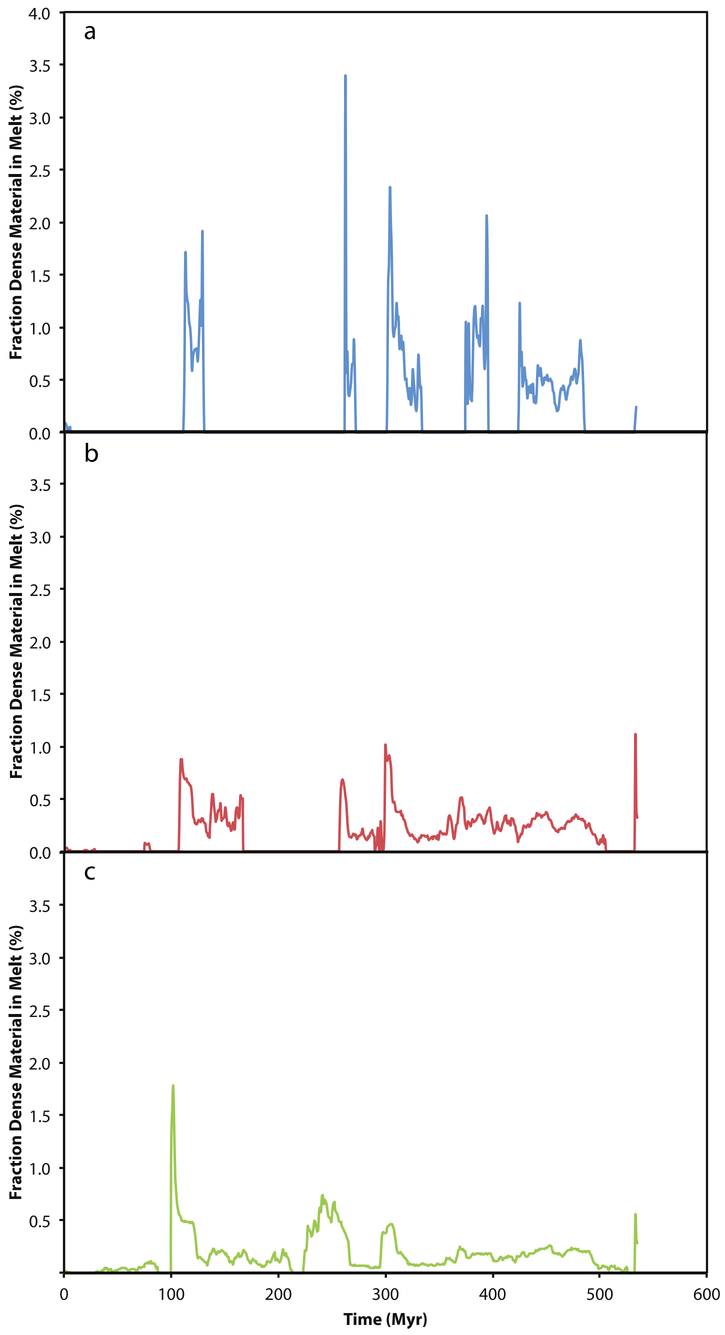

Supplementary Figure 1 Fraction of dense material in melt as a function of potential temperature across the mantle. **a** Temperature difference between hottest and coldest part of mantle is 3000. **b** Temperature difference between hottest and coldest part of mantle is 3500. **c** Temperature difference between hottest and coldest part of mantle is 4000.

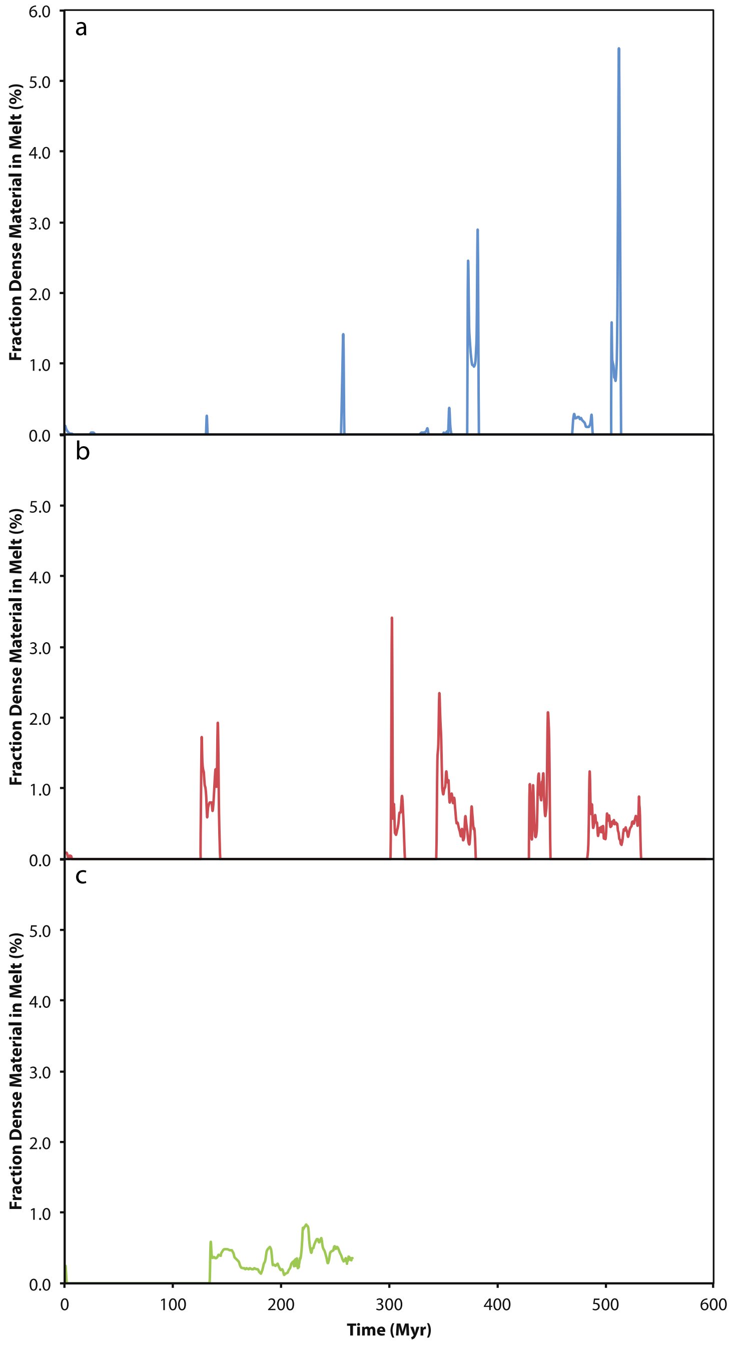

Supplementary Figure 2 Fraction of dense material in melt as a function of the activation coefficients associated with the temperature-dependent viscosity across the mantle. **a** Maximum viscosity contrast equals 1,000 times due to temperature. **b** Maximum viscosity contrast equals 10,000 times due to temperature. **c** Maximum viscosity contrast equals 100,000 times due to temperature.

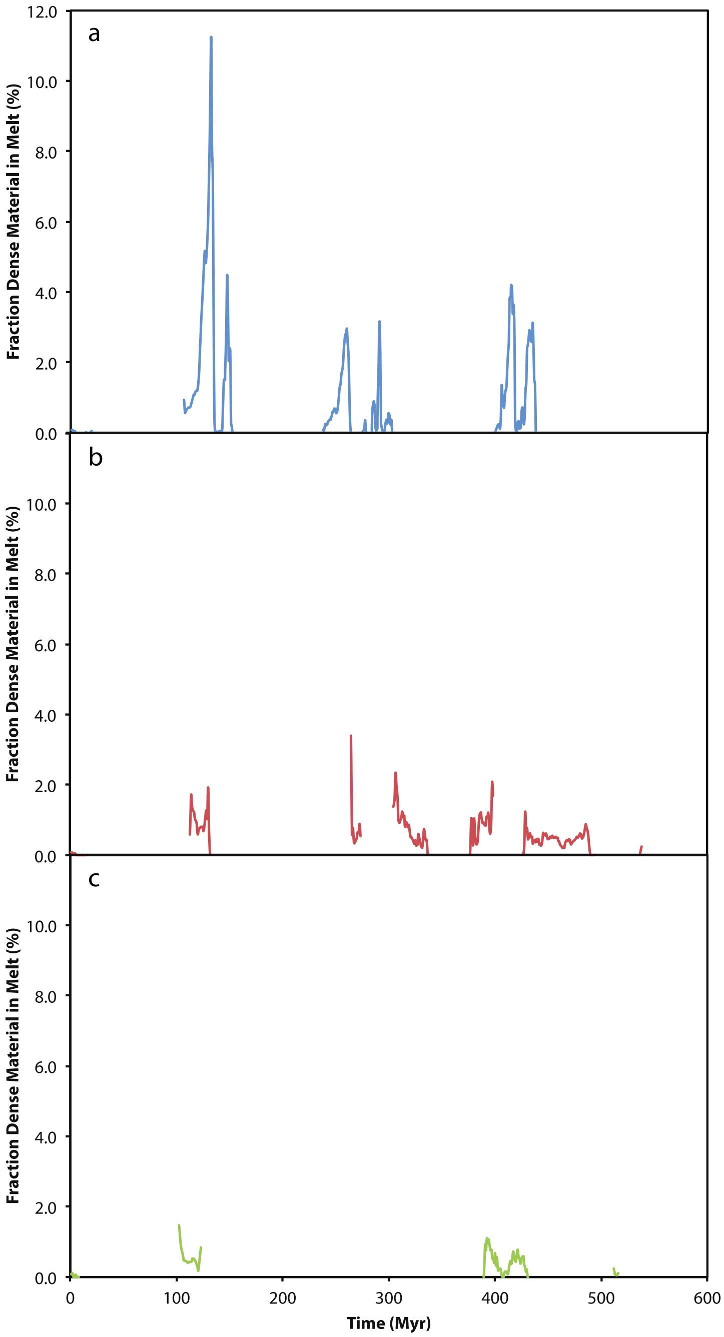

Supplementary Figure 3 Fraction of dense material in melt as a function of the buoyancy number (B) with a potential temperature across the mantle of 3000. **a** Non-dimensional buoyancy number equals 0.7. **b** Non-dimensional buoyancy number equals 0.8. **c** Non-dimensional buoyancy number equals 0.9.
